# On testing the strength independence assumption in retrieval-induced forgetting

**DOI:** 10.3758/s13423-015-0991-4

**Published:** 2016-01-11

**Authors:** Jeroen G. W. Raaijmakers

**Affiliations:** Department of Psychology, University of Amsterdam, Nieuwe Achtergracht 129B, 1018 WS Amsterdam, The Netherlands

**Keywords:** Human memory, Inhibition and memory, Statistics

## Abstract

Strength independence refers to the assumption that in a retrieval-induced forgetting paradigm, the increase in performance for the practiced items (RP+) is independent of the decrease for the related and supposedly inhibited items (RP−). One way in which this assumption has been tested is by examining the correlation over subjects between these two measures. The finding that there is no such correlation has been taken as evidence for the inhibition account and against noninhibitory accounts of retrieval induced forgetting. We report several, large-scale simulation studies using a simplified version of the SAM model (Raaijmakers & Shiffrin, *Psychological Review*, *88*, 93–134, 1981). The results clearly show that such a noninhibitory model is not likely to predict a significant correlation, despite the fact that on the level of the predicted probabilities such a correlation is clearly present. Additional simulations show that this is a very general result and not specifically related to the SAM model that was used. We conclude that such correlations do not provide a good test for the strength independence assumption and will not be able to distinguish between inhibitory and noninhibitory explanations of retrieval-induced forgetting.

One of the most controversial issues in current memory research is the question of whether forgetting may be due to a process of inhibition. According to a number of researchers (Anderson, 2003; Bäuml, 2008), interference-like forgetting is (mostly) due to a cognitive control process in which competing, but currently incorrect information is suppressed to be able to retrieve the target information. A standard paradigm to investigate this type of retrieval-induced forgetting (RIF) is the retrieval practice paradigm. In this paradigm, the participants first learn a list of category-exemplar pairs. Next, some of the items from some of the categories are given additional retrieval practice. Thus, there are practiced categories and nonpracticed categories (RP vs. NRP). In addition, from the practiced categories some items are given retrieval practice (RP+) and some are not given retrieval practice (RP−). According to the inhibition account, the RP− items will be interfering during the retrieval practice of the RP+ items and hence these will be suppressed or inhibited. The NRP items do not suffer from this inhibition. As a result, the RP− items will do worse on a final recall test compared with the NRP items. The inhibition or RIF effect therefore is typically measured as the difference between the NRP and RP− items.

Such an account runs counter to more traditional explanations of this type of forgetting, that are based on the notion that the observed forgetting of the RP− items might be explained by increased competition from the RP+ items. According to such noninhibitory accounts of forgetting, the decreased recall of the RP− items is due (at least in part) to the fact that the related RP+ items have been strengthened during the retrieval practice and hence these RP+ items are now interfering or competing more during the final recall test, and it is this increase in competition that is responsible for the increased forgetting of the RP− items.

There has been much debate in the recent literature about the relative virtues of these two explanations. Research has focused on testing specific properties thought to differentiate inhibitory from noninhibitory accounts based on competitive retrieval. However, no clear resolution has been achieved, primarily because the findings can often be interpreted in different ways and therefore do not clearly differentiate between the two accounts (Anderson, 2003; Raaijmakers & Jakab, 2013; Verde, 2012).

In this paper, I will focus on one such property that has been claimed as being fundamental to the inhibition account and as being inconsistent with competitive retrieval accounts, the so-called *strength independence* assumption. According to this assumption, the amount of inhibition is independent of the strength that is gained by the RP+ items during the retrieval practice phase of the experiment. This contrasts with the prediction from strength-based noninhibitory models, such as the SAM model (Raaijmakers & Shiffrin, 1980, 1981; Mensink & Raaijmakers, 1988). In such models, the factor that is responsible for the increased performance for the RP+ items also is the factor that is (at least in part) responsible for the decrease in performance for the RP− items, hence the increase for RP+ and the decrease for RP− would be expected to be correlated.

This strength independence assumption has been investigated in two ways. In the first type of experiment, two experimental conditions are created that show equal levels of RP+ recall but that differ in the size of the RIF effect, hence supposedly demonstrating that the size of the RIF effect is independent of the strength of the RP+ items (Anderson, Bjork, & Bjork, 2000). In the prototypical experiment of this kind, a comparison is made between two types of additional practice, the standard retrieval practice condition and a restudy condition in which the RP+ items are presented for another study trial. The standard finding is that performance on the RP+ items is about equal in both conditions, but there is a significant RIF effect only in the retrieval practice condition.

However, although this result seems like a strong confirmation of the strength independence assumption, it can in fact be accounted for by a noninhibitory strength-dependent model, as demonstrated by Raaijmakers and Jakab (2012). If one makes the reasonable assumptions that only retrieved items can be strengthened and that successful retrieval leads to a much more effective strengthening compared to an additional study trial, the observed dissociation between RP+ and the RIF effect can be easily accounted for. Basically, the idea is that during retrieval practice no feedback is given and this leads to a bifurcated distribution for the underlying strength distributions (Kornell, Bjork & Garcia, 2011) in which some of the strengths are very high (those for the items that were successfully retrieved) and some are low (those for the items that were not retrieved). In such a situation, the observed proportion correct in the RP+ condition is not a good measure for the underlying strength of those items, because vastly different strength values can lead to equal *average* recall probabilities.

More recently, another type of evidence has been proposed for the strength independence assumption. In these analyses, one looks at the correlation between the strengthening of the RP+ items and the size of the RIF effect. Such analyses have been reported by Hanslmayr, Staudigl, Aslan, and Bäuml (2010), Staudigl, Hanslmayr, and Bäuml (2010), and Hulbert, Shivde, and Anderson (2012). In the meta-analysis performed by Murayama, Miyatsu, Buchli, and Storm (2014), a positive correlation was obtained between the increase in performance for the RP+ items and the decrease obtained for the RP− items. However, this analysis included studies in which there was no control for output interference effects. That is, in the standard retrieval-induced forgetting paradigm, final recall may be tested either by presenting the category name with the instruction to recall as many of the presented category exemplars as possible, or by presenting the category name plus the first letter or letters of the target exemplar. In the latter procedure, the output position of a given item (and therefore the amount of output interference) is under experimenter control. When the analysis was restricted to those studies in which such output interference effects were controlled, there was no longer a relation between the strengthening of the RP+ items and the decrease for the RP− items.

The finding that there is a difference depending on whether output interference effects are controlled might be explained by the assumption that the (stronger) RP+ items often will be recalled before the RP− items and this will negatively affect the recall of the remaining items within the category (that is, the RP− items). In the meta-analysis of Murayama et al. (2014), this effect will be even stronger, because they included some studies in which participants were forced to recall the RP+ items before the RP− items (Murayama et al., 2014, p. 1389).

A problem with the meta-analysis by Murayama et al. (2014) is that the analysis was performed across experiments. Even if the competitive retrieval account is correct, it is not at all clear that experiments in which the mean recall of the RP+ items is relatively high (compared with the NRP items) would necessarily have to show a relatively large RIF effect. However, similar results have been reported by Hanslmayr et al. (2010), Staudigl et al. (2010), and Hulbert et al. (2012) using data from a single experiment. In these analyses, the increase in mean RP+ recall and the decrease in mean RP− recall were correlated over subjects. In all of these analyses, the results were similar: the correlations were small and not significant. For example, in Hulbert et al. (2012), the correlations were approximately 0.12 (using standard recall measures) or approximately 0.06 (using normalized scores, taking item differences into account).

In sum, the results do not seem to be in accordance with the predictions of strength-based accounts of retrieval-induced forgetting. However, as we mentioned in the past (Raaijmakers & Jakab, 2013, p. 112), one has to be careful in interpreting such null correlations since previous model simulations (Mensink & Raaijmakers, 1988) also have produced only weak correlations in paradigms where they should (theoretically) be present. We report a large-scale simulation study to determine to what extent a strength-based competition model will predict a substantial positive correlation between the facilitation of RP+ items and the decreased recall of the RP- items, as has been assumed by proponents of the inhibition account.

## Simulation model

A standard retrieval induced forgetting paradigm was simulated. In each simulated experiment, 32 “subjects” participated. The study list consisted of 16 categories of 4 items each. For any given subject, half of the items from 8 of the categories were given additional retrieval practice. Which categories were practiced was counterbalanced across subjects. For the final recall test, there were 4 scores for each subject each based on 16 items, the number of RP+ items recalled, the number of RP− items recalled and two NRP recall scores, of which one (NRP1) was the baseline for RP+ and the other (NRP2) the baseline for RP−. From these scores, we calculated the increase for RP+ (as RP+ minus NRP1) and the RIF effect (as NRP2 minus RP−). The latter two scores were then correlated to assess the relationship between the strengthening of RP+ and the size of the inhibition effect. The results that will be reported are based on 10,000 of such simulated experiments. All calculations were performed using Microsoft Excel using the PopTools package (Hood, 2010).^1^

### Initial study phase

Item strengths following the initial study phase were determined using a simple linear model (as in a standard ANOVA model). Specifically, the strength for item *j* in category *k* for subject *i* was calculated as:

$$ {S}_{ijk}=\mu +{\pi}_i+{\beta}_k+\pi {\beta}_{ik}+{\gamma}_{j(k)} $$

where μ is the overall mean, π_i_ is the effect for subject i, β_k_ is the effect for the category k, πβ_ik_ represents the interaction term for subject i and category k, and γ_j(k)_ is the effect of item j in category k. In this equation, π_i_, β_k_, πβ_ik_, and γ_j(k)_ are normally distributed random variables with mean 0 and standard deviations σ_π_, σ_β_, σ_πβ_, and σ_γ_, respectively.

For all of the simulations reported below, the following values for these parameters were used: μ = 150, σ_π_ = 0, σ_β_ = 20, σ_πβ_ = 10, and σ_γ_ = 20. The values were selected to ensure a large enough variability across subjects and items and to keep the predicted values for RP+, RP−, and NRP in the right ballpark (the absolute values are arbitrary, however, because they can be compensated by other parameters). Because in very exceptional cases the strength values might become negative (due to the fact that all the random variables were centered around zero), we imposed a minimum value of 1 for the strength values. The categories and items were then assigned to the subjects in such a way that each item occurred equally often in each condition.

### Retrieval practice

Recall probabilities were simulated using a simplified SAM model (Raaijmakers & Shiffrin, 1980, 1981; Raaijmakers, 2008). A detailed description of the model that was used can be downloaded at Raaijmakers (2015). Basically, the SAM model assumes that recall involves a series of retrieval cycles, each cycle consisting of a sampling and a recovery process. In the sampling process, a specific memory trace is selected with a probability that is a function of its relative strength to the retrieval cues. The recovery process involves the reconstruction of the name of the sampled item based on the retrieved features of the memory trace. The probability of successful recovery is a function of the absolute strength of the association of the target item to the retrieval cues. The number of retrieval cycles is assumed to be limited; that is, if the target item is not successfully sampled and recovered after Lmax cycles (where Lmax is a parameter of the model), the recall process ends with a retrieval failure. In this model, it is the sampling process that is responsible for the observed retrieval-induced forgetting: the increase in the strength of the RP+ items leads to a decrease in the relative strength of the RP− items and this increases the likelihood that the recall process for the RP− items ends in a failure.

There are two additional aspects of the SAM model that are relevant for the present application. The first is that after each successful recall, the associative strengths of the retrieval cues to the recalled item are increased (the so-called incrementing assumption). Thus, if an RP+ item was correctly recalled during the retrieval practice, its associative strength was incremented. The value of the increment was a normally distributed random variable with a mean of 90 and a standard deviation of 50 (a cutoff value of 0 was used to ensure that the increment could not become negative). The second is that additional retrieval cues lead to a focusing of the search process on those items that are associated to all of the retrieval cues. This is accomplished in the model by multiplying the associative strengths to the individual retrieval cues and using that product in the sampling equation. In the present simulation, that rule was applied whenever a recall test was given in which item-specific cues were used (i.e., the first two letters of the target item in addition to the category cue). In the present simulations, the strength of the item-specific cues to the target item was set to 1.0 and the strength to the other items was set to 0.5.

### Final recall

For the final recall, we used the same model as above with the exception that all strength values that originated from the initial study were multiplied by a forgetting constant *f* (set to 0.5), reflecting the forgetting between the original study episode and the final testing (e.g., due to context changes). In all other respects, the model was exactly equal to that used to generate recall probabilities for the retrieval practice phase. Scores for the RP+ items were obtained by summing the number of correctly recalled RP+ items from all categories, and similarly for the other recall scores (RP−, NRP1, NRP2).

## Results

**Fig. 1:**
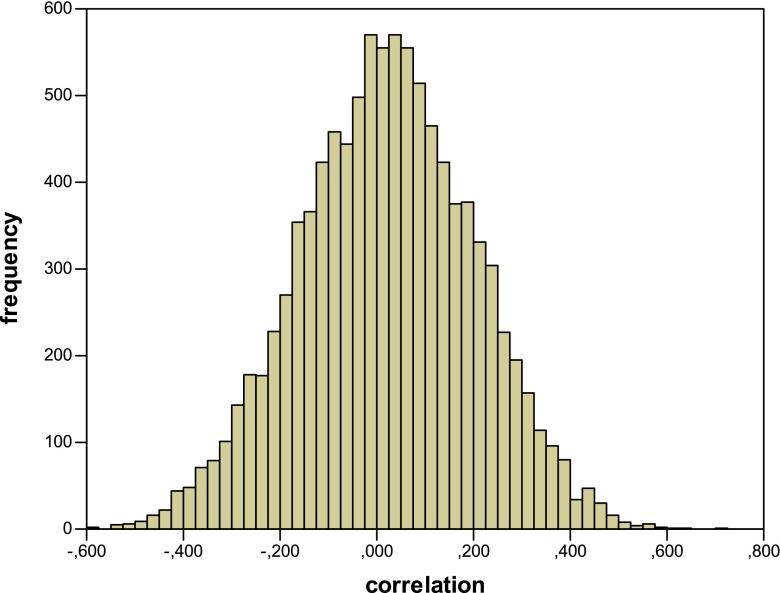
Frequency distribution for the correlation between the strengthening of the practiced items and the RIF effect for the model with variable increments (*Simulation 1*)

In the first simulation (*Simulation 1*), we used this model to generate data for 10,000 experiments with 32 “participants” each. We report the results in terms of the means and their standard deviations across the 10,000 experiments. The mean probability correct for the practiced items during the retrieval practice phase was 0.738 (s.d. = 0.020). For the final recall, the means were 0.769 (s.d. = 0.019) for the RP+ items, 0.539 (s.d. = 0.026) for the RP− items, and 0.641 (s.d. = 0.020) for the NRP items (we averaged the two NRP categories). Hence, the average practice effect for the RP+ items was 12.8 % and the average RIF effect was 10.2 %. The most important issue for these analyses was whether the model would predict a clear correlation across subjects between the size of the practice effect and the size of the RIF effect. For each simulated experiment, we correlated these scores for all 32 subjects. Figure 1 gives the frequency distribution for the obtained Pearson correlation coefficients. Somewhat surprisingly, the model does not predict strong correlations. The average correlation of all 10,000 experiments is just 0.020 with a standard deviation of 0.181. We also calculated how many of these experiments would have shown a significant correlation (using a one-sided *t*-test). This turned out to be a meagre 6.4 %. Hence, these model simulations provide no support for the idea that looking at such correlations will provide evidence for or against the strength independence assumption.

However, before drawing too strong conclusions from this simulation, it is advisable to see whether a modification of the model might lead to more substantial correlations. The most likely assumption that might be responsible for the lack of correlation is the assumption that there are no consistent differences between the simulated subjects in the size of the increment that is given to recalled items during the retrieval practice. Because the correlation is based on the average RP+ increment for a subject, this may decrease its range and hence the observed correlation. Hence, we ran a similar simulation study (*Simulation 2*) in which a single random value was sampled from the same normal distribution as before, but now this value was used for all recalled RP+ items for that specific subject.

**Fig. 2:**
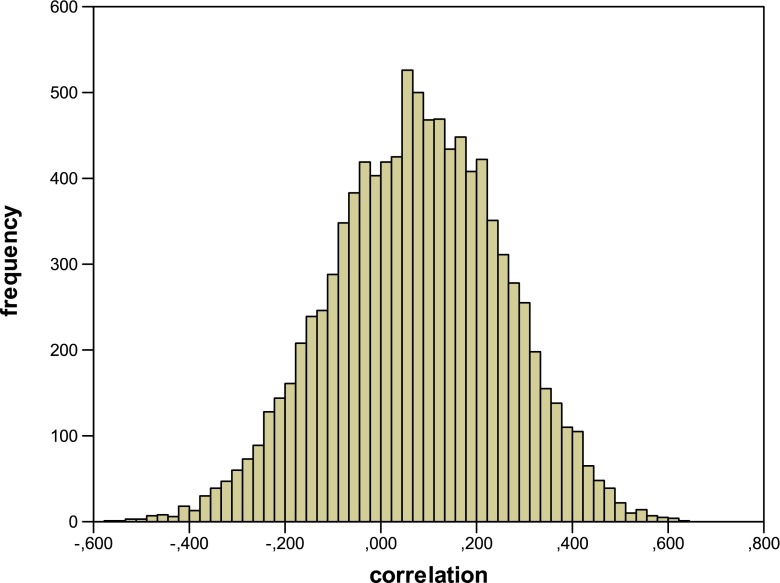
Frequency distribution for the correlation between the strengthening of the practiced items and the RIF effect for the model with constant increments (*Simulation 2*)

However, although this slightly increased the average correlation, it was still remarkably low: a mean correlation of 0.075 (s.d. = 0.179). Figure 2 gives the frequency distribution for the obtained correlation coefficients for this model. In this case, 11 % of the simulated experiments would have led to a significant positive correlation, which is indeed higher than was the case for the previous model but not much. The remaining scores did not change much from the previous simulation, with an average practice effect of 13.1 % and an average RIF effect of 10.1 %.

Apparently, there must be some other reason for the fact that the expected correlation does not materialize. It might be that the reasoning that led to this expectation is somehow flawed. With the next simulation, we will show that this is not the case. This simulation (*Simulation 3*) also will help to clarify what is really going on. In this simulation, everything was kept the same as in Simulation 2 except that at the final recall, we substituted the predicted recall probability for the actual recall scores. That is, in the simulations so far, the recall probabilities calculated from the model were used to generate a recall score for a specific participant and a specific item, i.e., either a 1 or a 0 score, indicating whether recall was predicted to be successful or not. In this simulation, the 1 or 0 score was substituted by the predicted recall probability. For example, if the predicted recall probability for a specific participant and item was 0.60, the recall score was set at 0.60 rather than at 1 (with probability 0.60) or 0 (with probability 0.40). Hence, the score for a specific subject for the RP+ items was determined not by counting the number of correctly recalled items but by averaging the predicted recall probabilities for the eight RP+ items, and similarly for the other item types. As before, the increase for the practiced items was calculated as RP+ minus NRP1 and the RIF score was calculated as RP− minus NRP2.

**Fig. 3:**
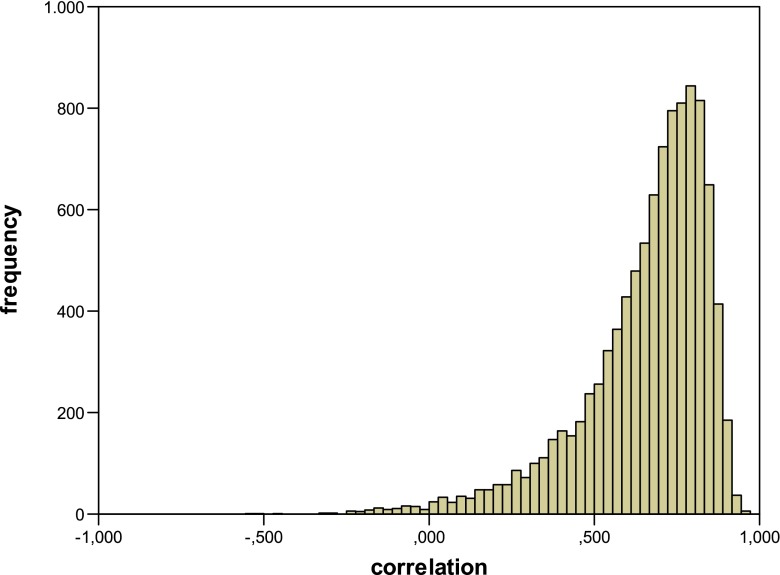
Frequency distribution for the correlation between the strengthening of the practiced items and the RIF effect for the analysis where probabilities are substituted for the actual recall scores (*Simulation 3*)

It turns out that this substitution does not change the average RP+, RP−, and NRP scores, but it does change the size of the correlation coefficient. The average correlation coefficient now jumps to 0.656 (s.d. = 0.193). The frequency distribution is given in Fig. 3, and it is clear that the pattern of the results is quite different from what we observed in the previous simulations. Based on these results, we may conclude that the model does predict a correlation between the strengthening of RP+ and the size of the RIF effect, but that it is very unlikely that a significant correlation will be obtained in an actual experiment (in which of course only the 1/0 data are available). Clearly, it is the variability that is introduced by the binary recall measure that is masking the correlation and is responsible for the results of the previous simulations.

A final set of simulations demonstrates that this is indeed what is causing this counterintuitive result. For this simulation (*Simulation 4*), we used a fixed value for the probability of recalling a NRP item at the final test (P(NRP) = 0.50), whereas the probabilities for the RP+ and RP− items varied in a linear fashion across subjects. That is, the probability of recalling a RP+ item was set equal to P(RP+) = P(NRP) + #Subj * 0.015 and P(RP−) = 0.6 – P(RP+) / 4 where #Subj is the index number for the subject (1-32). Hence, RP+ varied from 0.515 to 0.980 and RP− varied from 0.471 to 0.355, and these were perfectly correlated. These probabilities were then used to generate the actual recall data, i.e., the number of RP+, RP−, NRP1, and NRP2 items recalled using the same 16-category design as before.

Despite the fact that RP+ and RP− were completely dependent, the correlation between the increase for RP+ and the decrease for RP− was still quite low, with an average of 0.125 (s.d. = 0.176). Even in this highly artificial example, only 16.8 % of the experiments would have shown a statistically significant correlation. Clearly, the random variability in generating the data greatly reduces the magnitude of the correlation that might reasonably be expected.

The conclusion that seems to inevitable given these results is rather sobering: even if there is a strong relation between the strengthening of the practiced RP+ items and the size of the RIF effect, it will be virtually impossible to demonstrate such a relation in a typical retrieval induced forgetting study. Hence, the fact that several researchers have failed to find evidence for such a relation has little theoretical significance and certainly cannot be used as evidence against specific noninhibitory accounts for RIF effects.

## Discussion

Using several large-scale simulation studies, we have shown that models that in theory predict a clear relationship between the amount of strengthening of RP+ items and the decrease in performance for the RP− items are unlikely to generate a correlation between these two measures for the actual data of an experiment. The results of the last simulation demonstrate that the failure to obtain a correlation is not due to the specifics of the SAM model but that any probabilistic model that predicts such a correlation will lead to a similar result.

So what are the major factors that are responsible for the lack of a correlation? The final simulation shows that the most important factor is the fact that recall is inevitably measured using 1/0 scores. This factor introduces a substantial amount of noise that greatly masks the correlation. The second factor is that the correlation is taken between two difference scores, i.e., RP+ minus NRP1 and NRP2 minus RP−.

It is a well-known result from statistics that such difference scores tend to have low reliability, necessarily leading to low correlation with any other score (Lord, 1958; Bereiter, 1963). There are of course good and valid reasons for the use of these difference scores in this case, because a direct correlation between the RP+ and RP− would not work due to the confounding effect of interindividual differences (good memorizers will tend to have high scores on both RP+ and RP−). Hence, the use of difference scores hardly can be avoided, but one has to realize that it will affect the magnitude of the resulting correlation. The third factor is that there is a restriction of range issue. The average size of the RIF effect is not very large (especially when item-specific cuing is used) and rarely exceeds 10-15 %. Together these factors lead to the result that the observed correlation will be quite weak.

To further explore this latter issue, we also ran a set of simulations with the same setup as *Simulation 2*, i.e., using the SAM model with a constant increment for each simulated participant. However, we now chose the parameters in such a way as to increase the expected correlation. More specifically, we greatly increased the variance in the amount of strength stored for a specific participant (from 30 to 100) and we also increased the size of the mean increment applied after successful recall (from 100 to 150). In this way, some participants will receive many (large) increments while others will get only a few. However, this did not change the mean correlation very much: the average correlation was still only 0.073 (s.d. = 0.182). Clearly then, the factors leading to the low correlations are quite strong and difficult to change, even when the parameters are chosen to maximize the expected correlation. This may also be shown by a modification of the *Simulation 4* in which we used a setup with fixed and perfectly correlated probabilities for RP+ and RP−. In this simulation, we again fixed the recall probability for the NRP items at 50 % but now varied the probability of the RP+ items from 51.5 % to 98 % and the probability of the RP− items from 43.8 % to 6.6 %. As before, the RP+ and RP− probabilities were perfectly correlated. Although these values are highly unlikely in practice, the results are of interest because this setup should give the maximum correlation obtainable in this experiment. As one might expect, with these values we obtained a very large average RIF effect of 24.8 %. However, although the increased ranges did have an effect, the average correlation was still only 0.373 (s.d. = 0.144). Hence, for all practical purposes we may conclude that obtaining a sizable correlation is highly unlikely in this type of experiment.

What is crucial for this prediction is that there is a form of what might be called “local independence” between the recall of the items within a category. That is, even though there is a relation between recall of RP+ and RP− items over subjects, for any given subject the recall of the RP+ and RP− items are (locally) independent. For example, even though a specific RP+ item may have been strengthened quite a bit during the retrieval practice, leading to a high probability (e.g., 0.85) of recalling the RP+ item on the final test and simultaneously to a low probability of recalling the RP− item (e.g., 0.35), the probability that the RP− item will actually be recalled on a given trial is not affected by whether the RP+ item was or was not recalled on that same test trial. In a standard retrieval-induced forgetting paradigm such local independence is achieved through the use of item-specific cues (in addition to the category cue).

If no item-specific cues are present on the final test, local independence will no longer hold, because the stronger items will be recalled first leading to an additional decrement in the recall of the remaining items over and above the fact that their recall probability was already low. This explains why failure to control for output interference effects might produce a significant correlation between the strengthening of RP+ and the size of the RIF effect, whereas no such correlation will be present when output interference effects are controlled.

To check whether the SAM model would predict a correlation in such a category-cued recall test (with no item-specific cues), we ran a series of simulations with the standard SAM model for free recall. In the SAM model, output interference is due to (a) the increment for previously recalled items and (b) the assumption that as the number of retrieval attempts increases the likelihood of reaching the stopping criterion also increases. Both of these factors will lead to a decrease in the probability of recalling the later items. In these simulations, we used the same design as in the second set of simulations, i.e., with a fixed increment for each subject. We used the same model as before to generate sets of strength values that would be obtained after the retrieval practice phase. The only change from the previous simulations was that these values were then used as input for the SAM model for free recall. Due to the inherent complexity of the SAM model for free recall, we used 10 sets of strength values generated as before and each of these sets was used 10 times in the SAM simulation, leading to a total of 100 simulated experiments (again with 32 subjects and 16 categories per experiment). Note that with a single set of strength values, the outcome in the final test phase will still vary due to the variability in the retrieval process. Using this setup, we obtained an average correlation of 0.166 (s.d. = 0.168). As expected, the correlation is now larger than before, but it would still be significant in only 17 % of the cases. As mentioned before, in the meta-analysis of Murayama et al. (2014), a significant correlation was obtained when the analysis was restricted to experiments that did not control for output interference. These results are difficult to interpret, however, because the correlation was calculated across experiments rather than within a specific experiment.

Although we focused our discussion on the retrieval induced forgetting paradigm, the results presented have implications for other issues where the observation of null correlations has led to theoretical conclusions that may not be warranted. A prime example would be the independence of associative recall in the AB-AC interference paradigm (Greeno, James, DaPolito, & Polson, 1978). Greeno and colleagues interpreted the absence of a correlation between the recall of the B and C items as evidence against the unlearning assumption proposed by the Two-Factor Theory of forgetting. However, using a simulation of the SAM model, Mensink and Raaijmakers (1988) showed that such null correlations could be predicted by a model that (at least superficially) should have predicted a negative correlation (because recalling B should lead to a decrease in the probability of recalling C and vice versa). Mensink and Raaijmakers (1988) advanced several reasons that might explain the observed independence (including subject and item variability). However, based on the present results, it appears that the fact that recall is (inevitably) measured in terms of 0,1 scores also should have been considered as one of the factors that mask the predicted correlation, just as it does in the retrieval-induced forgetting paradigm.

## Footnotes

1 The files that were used for these and all following simulations are available for downloading at osf.io/4cf6h.
